# Clinical experience of thoracoplasty with absorbable rib substitutes

**DOI:** 10.1007/s00383-023-05413-1

**Published:** 2023-03-04

**Authors:** Juliana Mancera, Viviana Echeverri, Ana M. Castillo, Luis F. Rodríguez, Ricardo Zarama, Carlos Villamil, Juan P. Luengas

**Affiliations:** 1 Pediatric Surgical Oncology Department, Hospital Militar Central, Transversal 3° No. 49 – 00, Piso 8, 110231 Bogotá, Colombia; 2 Pediatric Surgery Department, Universidad Militar Nueva Granada, Bogotá, Colombia; 3 Pediatric Surgery Department, Clínica Pediátrica Colsanitas, Calle 127 No. 20-56, Bogotá, Colombia; 4 Pediatric Surgery Department, Hospital Infantil Los Ángeles, Carrera 32 No. 21ª-30, Pasto, Colombia

**Keywords:** Chest wall tumors, Thoracoplasty, Absorbable prosthetic ribs, Rib substitutes

## Abstract

**Purpose:**

Malignant chest wall tumors are rare in pediatrics. They require multimodal oncological treatment and local surgical control. Resections are extensive; therefore, thoracoplasty should be planned to protect intrathoracic organs, prevent herniation, future deformities, preserve ventilatory dynamics, and enable radiotherapy.

**Methods:**

We present a case series of children with malignant chest wall tumors and our surgical experience with thoracoplasty using absorbable rib substitutes (BioBridge^®^), after local surgical control. BioBridge^®^ is a copolymer made of a polylactide acid blend (70% L-lactic acid y 30% DL-lactide).

**Results:**

In 2 years, we had three patients with malignant chest wall tumors. Resection margins were negative, without recurrence at follow-up. We achieved good cosmetic and functional results, and no postoperative complications.

**Conclusion:**

Alternative reconstruction techniques such as absorbable rib substitutes provide protection, guarantee a flexible chest wall, and do not interfere with adjuvant radiotherapy. Currently, there are no management protocols in thoracoplasty. This option represents an excellent alternative for patients with chest wall tumors. Knowledge of different approaches and reconstructive principles is essential to offer children the best onco-surgical option.

## Purpose

Chest wall tumors represent a group of heterogeneous neoplasms which are rare in pediatrics; it´s estimated incidence is 1 per 1 million children and account for 2% of solid pediatric tumors [1–3]. They are usually malignant, and have a bone mesenchymal origin in 55% of cases. Ewing’s sarcoma is the most common histology [4]. Osteosarcoma represents 10–15% of tumors in this location; great proportion (75%) results from metastasis of long bones primaries [2]. Synovial sarcoma is even less common in the thorax; they account for 5–15% of all soft tissue sarcomas [1, 2]. According to Lopez et al. review, these are the most common histologies in children under 19 years who underwent chest wall resections for sarcomas [2].

Chest wall tumors are usually large volume and benefit from multimodal treatment, with neoadjuvant chemotherapy and local surgical control. The aim is to obtain negative resection margins and guarantee the patient a primary reconstruction. Resections are generally extensive with en-bloc resection of adjacent organs in the event of compromise. Variations in size and anatomic location of the post-resection chest wall defect are a major task. The challenge has been to develop a reconstructive strategy that offers adequate chest wall dynamics, avoids local deformity, and minimizes growth restriction [5]; therefore, prosthetic material for reconstruction must be chosen carefully.

The goals of reconstruction include protection, prevention of herniation and future deformities, preservation of ventilatory dynamics, and enabling radiotherapy [6, 7]. Traditionally, reconstructive options should be considered when the resection includes more than four ribs, the defect is greater than 5 cm, or involves anterior or sternal rib resections [5, 8]. With the increased availability of reconstruction materials, some surgeons propose the reconstruction of nearly all wall defects, intending to avoid the patient’s perception of chest wall instability [8].

The ideal prosthetic material for reconstruction will be a malleable, hypoallergenic, non-toxic, inert, and radiolucent material, that provides rigidity and allows the growth of the patient’s rib cage [5]. Since first chest wall reconstruction done by Tensini in the XVIII century, this field has evolved significantly [8]. Alternatives have emerged with the use of absorbable and non-absorbable prosthetic materials, without a consensus regarding the ideal material for this purpose [7, 9].

Prosthetic materials should provide rigidity, malleability, and radiolucency. The experience with mesh, cement, and metal implants described in the literature has its pros and cons [1, 7]; to mention: titanium osteosynthesis materials provide adequate protection; however, they limit the growth of prepubertal patients and therefore promote deformity. It was considered that titanium caused difficulties in the administration of radiotherapy, but currently, with precise radiotherapy simulation, the risk of refraction artifacts that generate greater toxicity is minimal. However, the technological equipment that allows this is not available in all centers, so it is not a factor to be neglected. Methyl methacrylate is a very rigid, impermeable, and radiopaque material that can limit ventilatory mechanics, cause pleural effusion and interfere with adjuvant radiotherapy [5, 7].

In consideration of the above, alternative reconstruction techniques have emerged and absorbable rib substitutes are especially useful in prepubertal patients [5]. After the first bio-absorbable material was successfully used in craniofacial surgery during the 90 s [5, 10], Tuggle et al. described their experience with the use of lactosorb copolymer in a series of pediatric chest wall reconstruction, with excellent biocompatibility [11–13]. This kind of copolymers have a notably firm consistency, are malleable when heated, and can be cut to shape. Furthermore, they withstand exposure to radiotherapy treatment [11, 14] and do not produce artifacts in radiologic examinations. On behalf of these properties, we consider them as an ideal prosthesis in the form of ribs substitutes. After skeletal stability is established, full tissue coverage can be achieved using autologous tissue, with different types of flaps depending on the extent of the resection [4].

Currently, there is no standardized protocol for chest wall reconstruction in pediatrics; therefore, each patient must be individualized [9]. Knowledge of different surgical approaches and reconstructive principles is essential to offer the patient the option that best suits their cancer treatment and generates the least possible deformity, with acceptable cosmetic results.

## Methods

We present a case series of three prepubertal adolescents with malignant chest wall tumors (Ewing sarcoma, osteosarcoma and synovial sarcoma). Inclusion criteria were patients younger than 18 years with a diagnosis of malignant chest wall tumor, diagnosed between 2010 and 2022, who were eligible for local surgical control according to the evaluation in images and stage of the disease. Thoracoplasty was done with absorbable rib substitutes in all patients. This is our surgical experience in chest wall reconstruction with absorbable rib substitutes (BioBridge^®^) after local surgical control. BioBridge^®^ resorbable rib substitutes are made of a polylactide acid blend (70% L-lactic acid y 30% DL-lactide) which maintains its strength within 18–24 months and is metabolized by hydrolysis; the stress loads are gradually transferred to the bone. Patient follow-up was performed according to the SIOP/COG protocols. The analyzed variables were survival, local or distant recurrence, chest wall morphology, and final esthetic and functional outcome.

## Results

### Case 1

**Fig. 1:**
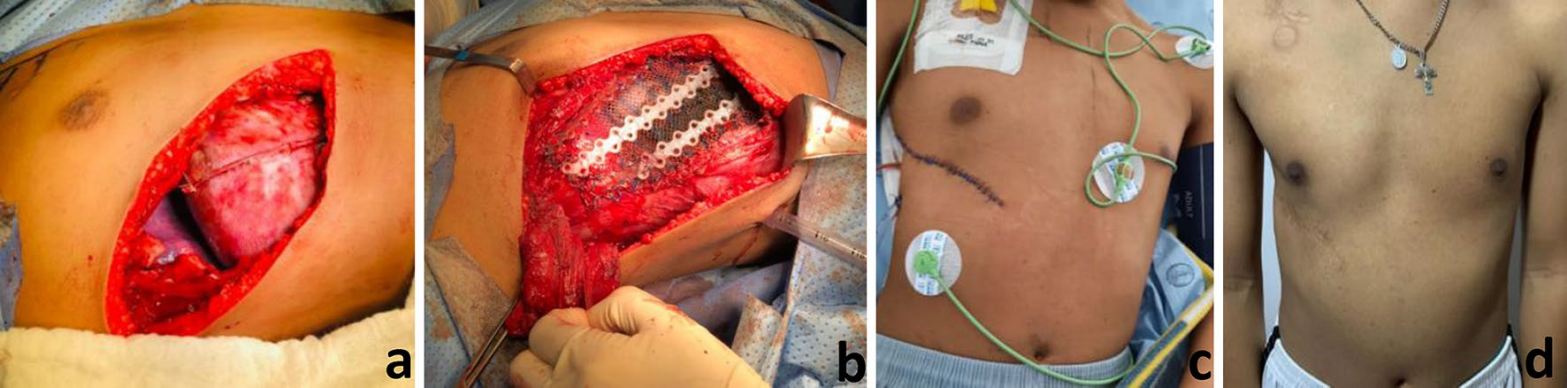
**a** Chest wall defect after en-bloc resection including residual fibrotic tissue in diaphragm and middle lung lobe. 1 cm Margins. **b** Primary reconstruction with a synthetic mesh and rib reconstruction with BioBridge^®^; soft tissue restoration with a latissimus dorsi flap. **c** Postoperative day 10. **d** 12 months after tumor resection and primary reconstruction

A 14-year-old patient with a 7-month history of respiratory symptoms and pleuritic pain, with subsequent appearance of a tumor in the right chest wall was presented. In the chest CT (computer tomography), we found a heterogeneous tumor with soft tissue density, dependent on the right anterior seventh rib, with dimensions of 12.5 × 9 × 6 cm, extending to the superior and inferior intercostal spaces and exerting a comprehensive effect on the diaphragm. Image-guided Tru-Cut biopsy confirmed Ewing’s sarcoma, positive for ESW/FLI1 translocation. Given the magnitude of the lesion and considering the potential area for resection, he received neoadjuvant chemotherapy. After the sixth cycle and a 98% reduction in tumor volume, local surgical control was performed. We found a 5 × 5 cm tumor dependent on the seventh right anterior rib, with adhesions to the ipsilateral middle lung lobe and diaphragmatic dome, which were resected en-bloc together with the anterior portion of the 6-7-8 costal arches, leaving a 1 cm margin (Fig. 1a). A primary reconstruction was performed with a synthetic mesh (absorbable and non-absorbable component) and rib reconstruction with BioBridge^®^; soft tissue restoration with a latissimus dorsi flap (Fig. 1b). The pathology reported hyalinized fibrous tissue with chronic inflammation without evidence of residual tumor or necrosis (complete response to neoadjuvant treatment), diaphragmatic dome, and pulmonary wedge negative for tumor. The patient received adjuvant chemotherapy, without the need for radiotherapy (Fig. 1c, d).

### Case 2

**Fig. 2:**
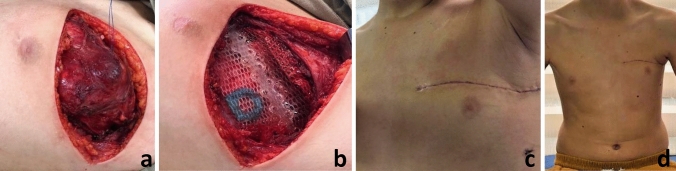
**a** 10 × 5 × 4 cm tumor dependent of the anterior portion of the third and fourth left ribs. **b** Primary reconstruction with a biosynthetic mesh and rib reconstruction with BioBridge^®^. **c** Postoperative day 8. **d** 1 month after tumor resection and primary reconstruction

A 13-year-old patient with a history of high-grade osteosarcoma in the left lower limb, who 18 months after local ablative control in the left extremity, presented the second primary in the left rib cage. The chest CT scan revealed a 10 × 5 × 4 cm tumor dependent on the anterior portion of the third and fourth left ribs. Image-guided Tru-Cut biopsy confirmed osteosarcoma. Neoadjuvant therapy was not administered; local surgical control was performed, finding a 9 × 5 × 4 cm tumor dependent on the described ribs, with involvement of the adjacent parietal pleura, without compromise of the lung or ipsilateral pectoral muscle (Fig. 2a). En-bloc resection was done, with 1 cm margins. We performed a primary reconstruction with a biosynthetic mesh and rib reconstruction with BioBridge^®^ (Fig. 2b). The pathology reported high-grade chondroblastic and osteoblastic osteosarcoma, with tumor-free resection margins. He received adjuvant chemotherapy (Fig. 2c, d)*.*

### Case 3

**Fig. 3:**
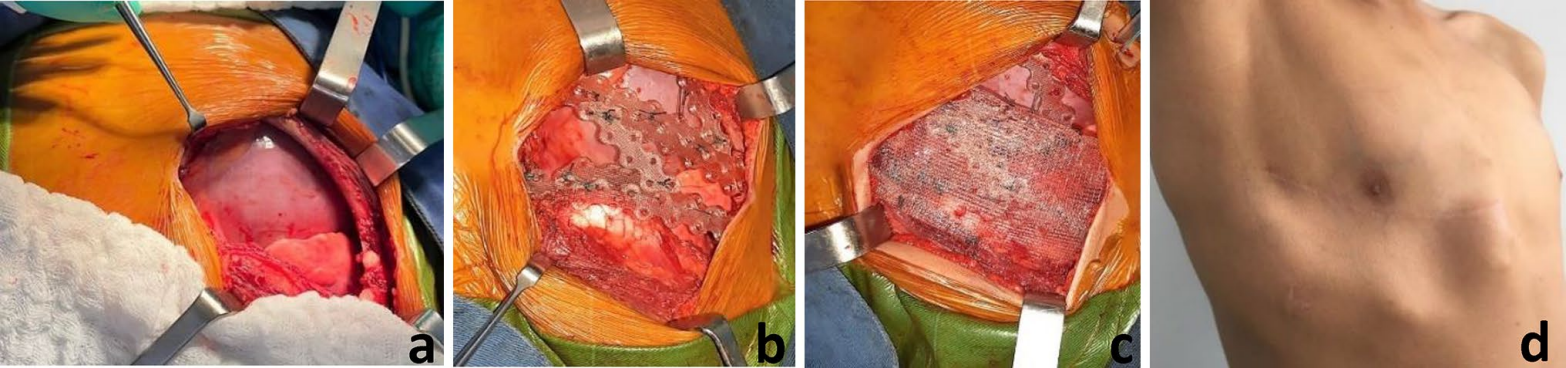
**a** Chest wall defect after en-bloc resection, guaranteeing 1 cm margins. **b, c** Primary reconstruction with a synthetic mesh (non-absorbable) and rib arches with BioBridge^®^. **d** 2 months after tumor resection and primary reconstruction

A 10-year-old patient with a 3-month history of a growing tumor in the right pectoral region, without associated symptoms. The chest CT showed a solid tumor of 3.8 × 4.8 × 3.8 cm, dependent on the fourth and fifth right costal arches, with extension to the pleura and superficial soft tissues. Image-guided Tru-Cut biopsy confirmed synovial sarcoma. The patient received a single cycle of neoadjuvant chemotherapy. In the local surgical control, a 5 cm tumor was found between the fourth and fifth right intercostal space at parasternal location. No lung involvement was identified. He underwent en-bloc resection, guaranteeing 1 cm margins, and primary reconstruction with a synthetic mesh (non-absorbable) and rib arches with BioBridge^®^ (Fig. 3a–c). The pathology reported synovial sarcoma with negative resection margins. Adjuvant chemotherapy was administrated; he did not receive radiotherapy (Fig. 3d).

Patients had a mean age of 12.3 years. En-bloc resection and primary chest wall reconstruction were performed in all cases. Resection included the surrounding soft tissues, biopsy trajectory, intercostal muscle, pleura, and any adjacent structures in which tumor invasion was suspected. Rib replacement for chest wall reconstruction was fashioned using BioBridge^®^ rib subtitutes. For each resected rib, the replacement strut was sutured to the bony component of the native rib with non-absorbable sutures. The plaques were cut to the size and the shape of the defect, and formed with heat. Microscopically tumor-free margins were obtained in all patients. No intraoperative or postoperative complications were detected.

Mean follow-up was 12 months, without detected recurrence. The final chest wall contour was symmetric and stable with no paradoxical movement. Concerning function, all patients can carry out normal daily activities including sports. We achieved a successful chest wall reconstruction with good cosmetic (Figs. 1d, 2d, 3d) and functional results, and no postoperative complications.

## Discussion

Malignant chest wall tumors require local surgical control, with wide resections and 1 cm margins. In our series, rib reconstruction with a titanium prosthesis and other rigid materials were not considered due to the projected growth of the patients and potential deformity. Alternative reconstruction techniques such as absorbable rib substitutes provide protection, guarantee a flexible chest wall, and do not interfere with adjuvant radiotherapy. Therefore, we decided to approach the thoracoplasty with absorbable prostheses (BioBridge^®^), in replacement of the resected rib portions, to give firmness and avoid a paradoxical respiratory movement.

In the presented three patients, we achieved a successful reconstruction of the chest wall, without postoperative complications and preserved ventilatory mechanics. The resection margins were negative. The chest domain and shape were maintained in all patients. The cosmetic results are good, they do not present scoliosis and there is no evidence of tumor relapse.

As these are uncommon tumors, the number of patients reported here is limited. However, important aspects of our experience are the age of the patients in a period of rapid growth. This reconstruction option represents an excellent alternative, since it avoids the deformity associated with the use of rigid materials, protects thoracic organs, and might not require new interventions to achieve an adequate functional and cosmetic result.

Currently, there are no management protocols in chest wall reconstruction. Absorbable rib substitutes represent an excellent alternative for thoracoplasty in patients with chest wall tumors. Knowledge of different surgical approaches and reconstructive principles is essential to offer children the best option that suits their cancer treatment.

## Data Availability

The datasets generated and/or analysed during the current study are not publicly available due to individual privacy, but are some general information is available from the corresponding author on reasonable request.
